# Navigating the human gene editing scene and proposing a new governance framework in Africa: from knowledge to policy to action

**DOI:** 10.3389/fgeed.2026.1866004

**Published:** 2026-07-29

**Authors:** Abdallah Borham, Fadya M. Elgarhy, Aalaa Moghith, Mohamed Tarek Elswefy, Rawan Ibrahim Mansour, May Bakr

**Affiliations:** 1 School of Sciences and Engineering, Institute of Global Health and Human Ecology, The American University in Cairo, New Cairo, Egypt; 2 Egypt Center for Research and Regenerative Medicine (ECRRM), Cairo, Egypt

**Keywords:** CRISPR-Cas9, ethical principles, governance structures, human germline genome editing, regulatory frameworks

## Abstract

Gene editing holds both hope and despair, necessitating vigilant, ethicolegal oversight to harness its advancement and avoid unintended effects like off-target effects and mosaicism. Despite efforts, governance fragmentation persists regionally and globally. Africa needs a phased, harmonized, continental governance framework for gene editing that combines regional oversight, national implementation, accountability, data governance, genomic sovereignty, and public participation. Policy harmonization is close to accomplishment, but obstacles persist. This paper proposes a practical continent-wide model anchored on African conformity, national legislative capacities, communal engagement, and data sharing. It presents an operational plan encompassing grassroots, nation-state, and regional regulatory bodies, centralized on a new pan-African agency for optimal oversight and governance. The model incorporates bioethical principlism and Ubuntu philosophy, forming the foundations of accountable and safe gene editing research and practice. Trespassing these foundations is a severe, inter-generational, and irresponsible act. This policy review critically analyzes the literature and evidence around gene editing, examining uncertainties, dilemmas, biases, and gaps in concepts. It presents an interpretation of the analysis in a narrative discourse with policy recommendations and governance reforms. The authors relied on empirical evidence and normative arguments. Gene editing is a multifaceted issue, yet a recourse for many patients with devastating diseases. Global progress has been hindered by procedural and operational defects and the controversy of enhancement versus therapy. Declarations, guidelines, and conventions should be prioritized to achieve universal benefits. A diligent consideration of collective risks is crucial to designing effective and inclusive policies. A multi-level approach is imperative for maintaining momentum and pace in technology development and localization.

## 1 Introduction

The capacity to rewrite the code of life has long been a theoretical aspiration of biomedical science. However, with the advent of high-precision gene-editing tools, the landscape of genetic engineering underwent a paradigm shift in 2012 with the CRISPR-Cas9 system: a tool celebrated for its cost-effectiveness, relative simplicity, and accessibility (Chan et al., 2015; Nelson and Gersbach, 2016). Human Germline Genome Editing (HGGE) stands at the brink of a new era in medicine, offering the seductive promise of eradicating severe monogenic disorders such as Huntington’s disease, cystic fibrosis, and beta-thalassemia before birth (Caval and iere, 2018; Human Genome Editing, 2017). HGGE expands reproductive options for prospective parents by allowing them to create a child biologically related to them without the risk of passing on inherited genetic disorders (Habermas, 2003). HGGE involves the deliberate modification of human embryos, sperm, or eggs, which could be for reproductive purposes or for research. As a result, any genetic alterations, whether beneficial, neutral, or harmful, are heritable and become a permanent part of the human gene pool, passing down to future generations. This distinct characteristic places HGGE at the center of one of the most contentious ethical and scientific debates of the twenty-first century (Lander et al., 2019). While the potential for therapeutic application is vast, the technical reality remains fraught with uncertainty (Baumann, 2016). In HGGE, two primary technical hurdles persist: off-target effects and mosaicism. These technical nightmares render the outcome of using this technology unpredictable, violating the fundamental bioethical principle of non-maleficence: “do no harm” (Heritable Human Genome Editing, 2020).

For years, the scientific community operated under a tacit generalized moratorium, agreeing that while *in vitro* research was valuable, clinical application (pregnancy) was premature. This fragile consensus was shattered in November 2018, when Chinese biophysicist He Jiankui announced the birth of the world’s first gene-edited human twin girls named Lulu and Nana, whose genomes had been modified to gain resistance to HIV. The experiment was criticized not only for its lack of transparency and informed consent but also for the lack of medical necessity, given that alternative, safer methods to prevent HIV transmission already existed (Cyranoski and Ledford, 2018). As reviewed by multiple scholars, it exposed the inadequacy of existing self-regulation and highlighted the dangerous gaps between technical capability and ethical oversight (Greely, 2019). Although some countries have already taken their position on HGGE, the pragmatic, sociopolitical, and ethical debates surrounding this technology are consciously growing (Baylis et al., 2020). HGGE provoked various debates about its value and probable downfalls (Almeida and Ranisch, 2022). Thus, the global scientific community proposed multiple moratoriums as an attempt to control the use of this technology (Lander et al., 2019; Lanphier et al., 2015).

Africa’s stance on the ongoing debate about HGGE remains mostly unclear, whether to adopt this technology for reproduction or not. This means unassessed needs, unknown capabilities, undiscovered capacities, and unclear intentions regarding this technology. To build this foundation from the ground up, it will require localized, state-nations-led, intercontinental momentum to pool resources, data, and political voice; this will lead to the initiation of an African HGGE initiative. Globally, efforts and policy dialogues have been ongoing throughout the last decade. Existing global frameworks structurally reproduce the marginalization they claim to address, and Africa’s regulatory moment with HGGE offers a rare window to build governance from the ground up (Baylis et al., 2020). Yet, this promise is inextricably bound to profound hurdles. Before that, the absolute question is why Africa needs a governance model for HGGE, and not to rely solely on global frameworks. The answer to this is that the commitment is mainly ethical and morally grounded. The obligation of nation-states to distribute resources and services with equity and justice is essential to decrease health disparities and induce citizens’ flourishing (Faure et al., 2021; Mwisongo and Nabyonga-Orem, 2016).

The global HGGE governance frameworks allegedly address equity concerns; they are structurally designed by and for high-income regulatory contexts (World Health Organization, 2021). This manuscript argues that existing frameworks perpetuate the very fragmentation they claim to remedy, and that Africa’s current regulatory opportunity with HGGE opens the door to building governance architectures by principles of continental equity, epistemic justice, and self-determination.

Considering these complexities, this paper aims to provide a distinctly African HGGE governance model, anchored in continental coordinating mechanisms, recognition of African knowledge systems, and participatory policy design. Therefore, simply adopting Western-centric models is problematic. We need thoughtful and innovative approaches to prevent the resurgence of global health inequities in this genomic era. After a comprehensive review of the current state of human germline genome editing, we analyzed the persistent technical barriers, mapped the fragmented global regulatory environment, and critically examined the ethical frameworks needed to govern this technology as well as the policy arena governing it. By synthesizing recent developments and global experiences, we argue that purely technical solutions are insufficient; a robust, continental ethical governance framework is urgently required before humanity takes the irreversible step of editing its own evolution.

## 2 Methodology

This study was conducted as a narrative policy review. We followed a collaborative process, assigning different sections to the authors. Each author conducted an independent review of the assigned literature, and the final manuscript was collectively evaluated to ensure consistency, coherence, and comprehensive coverage of the topic.

A search strategy was applied to identify the relevant literature that tackled the HGGE dilemma in terms of untapped uncertainties, hauling risks, governance issues, and proposed policy reforms. The inclusion criteria were the following: (1) focus on human germline genome editing; (2) publication in peer-reviewed journals or authoritative public health reports; (3) studies published in the last 15 years; and (4) articles in the English language only. The exclusion criteria were the following: (1) non-human germline genome editing and irrelevant articles; (2) non-peer-reviewed sources; and (3) articles in languages other than English. 200 articles were initially retrieved from PubMed, Google Scholar, Scopus, and Science Web databases. Also, reports from international organizations were included to cover this policy-related topic from non-scholarly perspectives. Concepts and keywords related to the research topic were used, such as human germline genome editing, African regulatory agencies, CRISPR-Cas9, global governance, Global and regional regulatory landscapes, and off-target effects. After removing duplicates and conducting title and abstract screening, several articles were excluded due to irrelevance, methodological limitations, or lack of required data. The remaining 55 articles were thoroughly reviewed and included in the final analysis.

While this narrative policy review synthesizes governance frameworks and evidence across multiple regional contexts, selective citation bias is a potential limitation. We acknowledge the risk of selective citation inherent in narrative reviews. To reduce this risk, we used a pre-specified search criterion across multiple databases, primarily PubMed and Google Scholar. We also conducted dual independent screening, actively searched for contradictory evidence, and purposively sampled across geographic regions to lower regional bias.

## 3 Background

### 3.1 HGGE risks and ethical foundations

HGGE’s technical complexity, including irreversible germline modifications and currently irreducible off-target effects, creates governance imperatives distinct from somatic cell therapies. Concurrently, the enhancement/therapy distinction, though scientifically unstable and culturally constructed, remains the boundary most governance frameworks attempt to police. We argue that these technical realities, combined with Africa’s historical exclusion from governance norm-setting, necessitate structures designed specifically to manage uncertainty under African ethical and regulatory conditions. The prospect of HGGE presents a profound technical and ethical dilemma that challenges foundational bioethical principles, social values, governance structures, and intergenerational responsibilities.

A central ethical challenge is consent and individual autonomy. Adult patients can provide informed consent for somatic interventions, but germline genome editing, especially at the embryo stage, affects future children and generations who are unable to consent (Evans, 2021). Altering a person’s genome retroactively before birth is an irreversible decision that disqualifies self-determination regarding a fundamental biological aspect (Robertson, 2003). The moral legitimacy of parental consent is further questioned when interventions go beyond disease prevention to enhance traits or when long-term risks are poorly understood (Almeida and Ranisch, 2022; Savulescu and Kahane, 2009; Brokowski and Adli, 2019). The principles of non-maleficence and beneficence, and their application to HGGE, are uncertain. Interestingly, ethical perspectives on therapeutic gene editing suggest that many consider it morally acceptable when safety and efficacy are established (Joseph et al., 2022; Gyngell et al., 2017). The somatic/germline barrier, once a moral barrier, now lacks strong alternatives, risking a “slippery slope” to non-therapeutic enhancement and widespread eugenics (Evans, 2021; Mulvihill et al., 2017). Therefore, HGGE presents a significant technical and ethical conundrum that necessitates a resolute, proactive approach that harmonizes local and regional realities with global traditions.

### 3.2 Global governance landscape and gaps

#### 3.2.1 Global analysis

Currently, there is a need for a well-structured governance mechanism that controls HGGE research. The current landscape is fragmented, largely voluntary, and lacks a robust global consensus. On a global level, in 2019, the WHO advisory committee on human genome editing agreed to develop a structured governance structure to ensure ethical human genome editing research (World Health Organization, 2025). In 2021, the WHO published the governance framework for HGGE, which addressed both procedural and substantive values to ensure the proper use of this advanced technology. The framework proposed multiple areas that outline the HGGE application: leadership by WHO, fostering international collaboration for setting the governance structure and oversight, establishing registries for HGGE clinical trials, enacting research jurisdictions with proper oversight mechanisms, a mechanism for confidential reporting of malpractices, promoting equitable access for HGGE and licensing practices, inclusion of global dialogue for education and empowerment, establishing clear ethical values and principles, and finally reviewing the implementation of these recommendations in 3 years. However, no assessment report has been published till the end of 2025 (World Health Organization, 2025). So far, there have been 48 clinical trials registered since 2021 in the WHO registry for clinical trials that have flagged gene editing in the text submitted, out of a total of 93 research studies registered since 2015 (World Health Organization, 2026).

On the other hand, UNESCO’s International Bioethics Committee called for a global dialogue to ensure human dignity and avoid altering human heredity. Additionally, the International Commission on Clinical Use of Human Germline Genome Editing (2020) declared that HGGE is still in early stages for clinical application and provided robust conditions for future clinical trials (International Bioethics Committee IBC, 2021).

On the national level, many countries have set up national policies in this regard, yet these policies widely vary. Some countries adopted strict bans, such as those in the EU via the Oviedo Convention (International Bioethics Committee IBC, 2021), which prohibited germline modification for reproductive intent. Additionally, Germany, France, and Italy banned embryo modification with strict criminal penalties. Other countries developed a set of regulations without complete prohibition, such as the UK, that allows embryo editing for research only under strict oversight, while clinical use is still banned. On the contrary, in the USA, it is permitted but highly regulated (Genetic Literacy Project, 2020). Nevertheless, most countries lacked comprehensive public engagement in regulating HGGE implementation, especially those marginalized, underprivileged, or with specific cultural or religious backgrounds. No defined balancing of demographic diversity and viewpoints was disclosed, as well as the absence of logistical funding issues (Conley et al., 2023).

#### 3.2.2 Governance gaps identified through systematic analysis of African HGGE readiness

The disconnect between global ambition and local reality led to a weakened, fragile governance structure with critical vulnerabilities. These gaps can be classified into three broad categories: foundational gaps, oversight gaps, and societal gaps.

Firstly, foundational gaps include the lack of common rules, ethical principles, and safety thresholds. Having no binding international law gives space for research misconduct (Shozi and Thaldar, 2023). In addition, scientists still disagree on a unified definition of “enhancement” versus “therapeutic use”, which is a core distinction for ethical application (World Health Organization, 2021). Moreover, safety thresholds and standards for assessing off-target effects and long-term consequences are discordant and ill-defined universally.

Secondly, oversight gaps involve the weak transparency, accountability, and enforceability of regulations. Traditional ethics committees are ill-equipped and lack the necessary capacity to oversee privately funded and multinational gene editing research (Major et al., 2024). Additionally, the WHO’s clinical trial registry is voluntary, and no global, independent auditing system exists to verify compliance. Many countries lack defined, clear criminal penalties, licensing systems, or obligatory reporting mechanisms.

**FIGURE 1:**
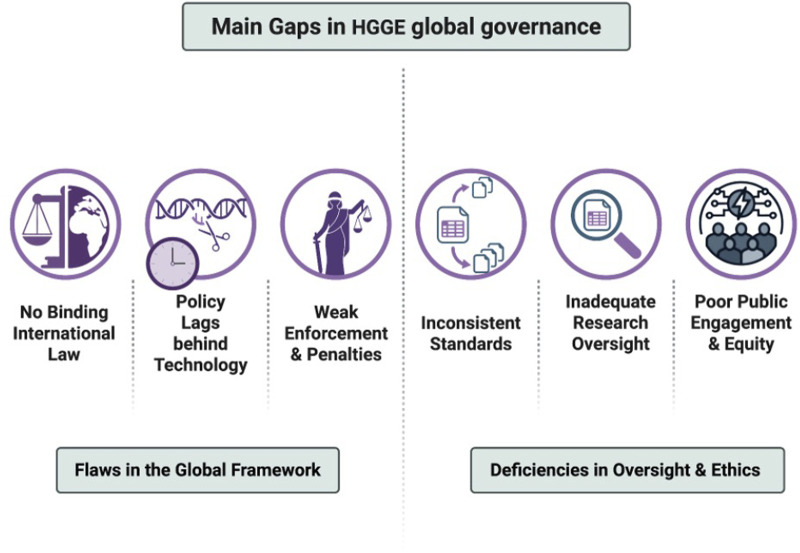
The main gaps in the HGGE global governance framework.

Thirdly, societal gaps involve the voices of the public not being considered within the policy-making processes. Moreover, there is a substantial risk that only wealthy people will benefit from this technology, which in turn exacerbates global inequalities (Conley et al., 2023). Finally, the rapid pace of technological discoveries outruns the slow pace of policy creation, resulting in outdated regulations, as shown in Figure 1, which summarizes the gaps in a graphical manner (Kannan and Najjar, 2020).

A higher-level reasoning of the governance gaps could take a second-order complementary categorization. What persists regardless of the classification is that the gaps undermine both the democratic legitimacy and practical effectiveness of HGGE governance frameworks at national and international levels. By surgical criticism and analytical dissection, a three-level literature-based examination of the gaps is presented.

At the discursive level, a parallel and equally consequential gap has emerged in how HHGE is publicly debated. Martani and colleagues argue that current discussions around HGGE have undergone a form of argumentative narrowing, a mode of framing that decreases the space for open moral deliberation, promotes the desirability of HGGE, and depoliticizes the issue. Drawing on the philosophical theory of technomoral change, which unravels the reciprocal influence of technology and morality, the authors demonstrate that this narrowing occurs as evolving biotechnologies quietly reshape moral norms before societies have had the opportunity to negotiate them openly, and they conclude that because of this mutual influence, a repoliticization of the discussion on HGGE is needed (Thaldar et al., 2021). This framing concern is particularly consequential for governance because, as the same authors note, if broad societal participation is a precondition for decision making on this issue, the public debate must do justice to the complexity of HGGE and cover the wide spectrum of interests, values, and most importantly, the principles at stake, which that logical narrowing structurally prevents from being achieved (Martani, 2024).

At the procedural level, the South African case illustrates how a substantively strong regulatory framework can collapse when, beneath, it is procedurally fragile and politically contested. The National Health Research Ethics Council’s (NHREC) 2024 guidelines established a regulated pathway for HGGE research and anticipated the possibility of clinical application, yet their adoption was weak, with consultation confined to research ethics committees and excluding broader scientific and public engagement (Kobayashi et al., 2022). This lack of inclusivity left the guidelines politically vulnerable, and when academic criticism focused on the fact that the guidelines contemplated the eventual possibility of children being born with edited genomes, those critiques gained traction and ultimately contributed to the NHREC’s decision to revoke the guidelines in July 2025. The South African episode thus demonstrates a foundational governance deficit that is by no means unique to one jurisdiction. The absence of participatory procedures capable of withstanding political contestation means that even technically and ethically sound regulatory instruments remain fragile and susceptible to instrumentalization by competing institutional actors (Thaldar et al., 2025).

At the empirical level, a third gap compounds both above: the persistent absence of large-scale, population-representative public opinion data from the Global South. While international surveys have explored public attitudes toward HGGE, especially in high-income countries, there is a lack of large-scale empirical data from Africa (McCaughey et al., 2016). In an applaudable attempt in South Africa, scholars have set a course for gathering evidence based on deliberative public engagement to examine public perspectives. Pillay and Thaldar published a protocol for a cross-sectional survey planning to capture public opinion in a format that enables direct comparison with deliberative findings. Based on the protocol, the study aims to generate a secondary dataset for evaluating AI-assisted qualitative analysis, reflecting the growing methodological innovation needed to handle the complexity and scale of public opinion research in this domain (Thaldar et al., 2021). The absence of such research is not merely a methodological shortcoming; it means that governance bodies making consequential decisions about the permissibility and conditions of HGGE research and clinical application are doing so without knowing whether their frameworks reflect the values and priorities of the populations most directly affected, including those bearing the highest burden of genetic diseases that HGGE could potentially address (Thaldar et al., 2026). Taken together, these three interrelated gaps: discursive narrowing in scientific and policy discourse, procedural exclusion in regulatory design, and, in addition, the empirical deficit in public opinion data from LMICs contexts, produce risky governance frameworks that are democratically illegitimate, normatively distorted, and empirically ill-founded.

To address these compounding deficiencies, a triplex strategy is proposed. First, and as mentioned above in earlier sections, regulatory bodies should institutionalize multi-stakeholder consultation mechanisms to encompass civil society organizations, disability rights advocates, patient communities, and populations most likely to be affected by the genetic conditions HGGE may target, thereby building the procedural legitimacy that the South African case demonstrated to be indispensable for regulatory durability (Thaldar et al., 2025). Second, scholars, science communicators, and decision makers should actively work to repoliticize the HGGE debate by creating deliberative spaces that surface categorical, sociopolitical, and equity based concerns rather than defaulting to technocratic risk-benefit framings; as Martani and colleagues propose, undoing discursive narrowing requires conscious effort to reopen questions about whether the further development of this technology is morally desirable, questions that have been progressively seized as the debate has shifted toward the technical management of an assumed inevitability. Third, sustained investment is needed in large-scale, cross-sectional, as well as longitudinal public opinion research, particularly across the African context, to generate the empirical baselines without which regulatory decision-making will be unreliable (Martani, 2024).

### 3.3 Structural drivers of governance failure: the He Jiankui case as governance indicator

After the 2018 scandal, the global regulatory landscape has been revealed as a patchwork of divergent policies, reflecting big cultural and legal differences. On one end of the spectrum lies the “precautionary approach,” exemplified by the Oviedo Convention (Andorno et al., 2020; Kevles, 2009; Council of Europe COE, 1997). Conversely, nations such as the United States have adopted a more procedural blockage; while there is no explicit ban on the technology itself, legislative riders currently prevent the FDA from reviewing applications for clinical trials involving heritable genetic modifications, effectively creating a moratorium. The 2018 CRISPR babies case reveals not merely an ethical lapse but systemic governance vulnerability. Competitive nationalism in science, regulatory arbitrage and permissive jurisdictions, prestige economy of ‘firsts,’ and inadequate institutional review oversight. These structural conditions exist in modified forms across the globe and specifically in contexts with nascent HGGE regulatory infrastructure. Understanding these drivers, rather than treating the case as an isolated scandal, sharpens the design of governance mechanisms that address root causes rather than symptoms (Nuffield Council on Bioethics, 2018). This event, widely condemned as “irresponsible” and “unethical” by the international scientific community, served as a wake-up call. The root cause of this incident, as the literature suggests, is mainly the weak formal constraints in China, which had, back then, no legal barriers, only an academic advisory system presented. Owing to the enforcement of competitive nationalism in science that China adopts, and as a perceived Chinese elite scientist, He Jiankui took a risk in pursuit of state prestige and incentives. Lastly, the prestigious environment of firsts is widely dominant in the scientific arena; an achievement such as gene-edited babies represents a career-defining triumph. Thus, ethical consensus alone is insufficient; governance frameworks are vital to address and prevent such an affair (Greely, 2019; Alonso and Savulescu, 2021).

Furthermore, a critical review urging an African perspective on germline genome editing highlights that discourses shaped in Western bioethics may not fully capture values and concerns in other regions, which raises questions about global justice and equity in policymaking (Shozi and Thaldar, 2023). Thus, not only is equitable access central, but also inclusive, culturally sensitive deliberation about acceptable uses, governance mechanisms, and global equity is essential. As a result, and drawing from the Chinese model along with global milestones achieved in this field, a pan-African governance awakening is urgent. The model should consider global incidents that violated ethical standards and scientific professionalism to serve as a reproductive oversight initiative for the continent.

## 4 Discussion

### 4.1 Justice as a central pillar for good governance practice

Justice serves as a building block for an inclusive governance model. The justice framework presented here is built upon four fundamental pillars: Distributive, recognition, epistemic, and procedural. A just HGGE governance for Africa requires attention to these justice dimensions, as each implies distinct governance mechanisms. Fair distribution of benefits and burdens raises critical societal concerns (Walker, 2026; Deplazes-Zemp, 2019).

Africa-specific distributive injustice is central to a low amount of research and inclusion in studies and trials that would create local, reliable data and induce innovation that is both domestic and sovereign. Some scholars argue that HGGE will eliminate health inequities caused by genetic conditions like Sickle Cell Disease, which disproportionately affects African populations (Lee and Sawai, 2024). Access to germline gene editing technology is highly inequitable. Current and near-term gene editing therapies are likely to be expensive and available mainly in affluent countries or to wealthy individuals. This raises fears of a genetic divide, where only privileged segments of society can afford to eradicate or reduce genetic diseases, and possibly enhance traits, exacerbating social inequality (Baylis, 2019). This could create a societal divide between “Gen-Rich” (enhanced) and “Naturals” (not enhanced) groups, with enhanced individuals inheriting increasing socioeconomic privileges due to their health advantages (Silver, 1998; Coller, 2019). Communities in Africa face a significant risk of injustice, particularly in healthcare. This is because decision-making processes seldom involve patients, which underscores the need for ethically vigilant systems and individuals (Halley et al., 2023). Burden distribution injustice implies that any unintended adverse effects may disproportionately affect future generations. Critics warn that this not only will infringe upon individual rights but also jeopardize the genetic legacy of the entire human species (Almeida and Ranisch, 2022).

When it comes to recognition and epistemic justice, ethics recommendations and guidelines tend to obscure the values and philosophies of the individuals to whom this technology is applied. In the African HGGE context, it is crucial to recognize that concepts and values rooted in local intuitions and dominant ways of life are primary contributors to the knowledge production that underpins frameworks applied to genetic challenges (Ewuoso et al., 2022). Building on Ubuntu ethics, which emphasizes community interests and integrates past and future generations, straightforwardly justifying equitable access to HGGE, supported by governments and regional/continental coalitions (Thaldar et al., 2022). Genomic sovereignty is indeed vital; however, alone, equity and justice are questionable. Rather, developing governance tools would ensure fair distribution of benefits among African populations and researchers (Kabata and Thaldar, 2023). Therefore, adopting principles of fairness, solidarity, reciprocity, and equitable participation in knowledge production, use, and circulation will lead to reforms in genomic research that will provide and enhance genomic resources.

Procedural justice is centered on the inclusiveness of policies and frameworks and making them subject to public scrutiny. Most importantly, it solves the dilemma of who makes decisions about policy and programmes whilst showcasing whose interests are most served by those decisions (Cavaliere et al., 2019). In the African context, fragility and exclusion are seen as there has been little to no attempt to engage the public in discussions on the perimeters of the technology (Thaldar et al., 2025; Jasanoff et al., 2019; Ssebunnya, 2023). Thus, there are prerequisites for procedural justice pertaining to HGGE governance and oversight (Iltis et al., 2021). Inclusive stakeholder participation, deliberative democratic models, and structured community engagement are required when accountable governance is sought (CAVALIERE et al., 2019). The concept of “Citizens’ Juries” offers a promising participation model by engaging lay persons provides dual benefits of both normative public perspectives and political guidance on HGGE. Initiatives like H3 Africa Consortium have successfully utilized consultative models to allow open discussions to address ethical questions, highlighting a potential momentum (Tindana et al., 2019). However, questions like what constitutes a diverse stakeholder group should still be discussed, even though a multi-origin representation is known to be more beneficial than scientist-dominated forums. Research ethics committees’ capacity strengthening is required to enhance the ethicolegal deliberations, especially on the institutional levels, which will serve the governance reform action, continent-wide (Hyder et al., 2015). As a result, mature discussions on financing and funding will arise as a clear objective and an outcome of the previous corrective actions led by the institutions and the people representing the communities in totality. Evidently, for these reasons, justice is the cornerstone of governance (Tindana et al., 2019).

### 4.2 African ethical foundations for HGGE governance: beyond principlism

While this manuscript draws on bioethical principlism (beneficence, non-maleficence, autonomy, justice), we argue that HGGE governance designed for African contexts must ground itself in African philosophical traditions. Ubuntu is understood as the ethics of relational interdependence, collective wellbeing, and intergenerational responsibility (Shozi and Thaldar, 2023). It offers a distinctly different framing for African HGGE policy and regulatory efforts. Consequently, its implications on consent will draw different extrapolations than Western principlism; autonomous individual consent is a prerequisite. Ubuntu’s relational ontology suggests consent should be understood as a thoughtful communal consultation embedded in kinship and community structures (Anye-Nkwenti Nyamnjoh, 2025; Staunton and de Vries, 2020).

Operationally, this implies HGGE governance mechanisms should include: (Chan et al., 2015) community deliberation processes alongside individual consent; (Nelson and Gersbach, 2016) recognition of extended family authority in germline decisions not just individual couples; (Caval and iere, 2018) intergenerational consultation mechanisms, and how this could affect future generations’ autonomy (Munung et al., 2022). Comparatively, Western framing of intergenerational justice revolves around who gets access in the present time, mostly distributive. Meanwhile, Ubuntu holds relational continuity intact across time as it emphasizes the interconnectedness of generations, which again implies that HGGE should be evaluated *a priori* for its effect on descendants’ choices (Thaldar et al., 2025).

Institutionally, this suggests mechanisms for: (Chan et al., 2015) mandatory intergenerational review; (Nelson and Gersbach, 2016) reversibility assessment, where possible; (Caval and iere, 2018) long-term community benefit-sharing, meaning that modifications should benefit multiple future generations, not just immediate users. Hence, Ubuntu philosophy explicitly showcases the distinct variation from systems focused solely on current access; western ethicolegal architecture (Imafidon, 2022).

Eventually, the distinction of communal benefit-sharing ideologies is quite discernible between the two ethical schools. As Ubuntu exhibits the collective flourishing frame, in contrast with the Western framing of individual risk-benefit portrayals. Critical learning would be that: (Chan et al., 2015) benefits should flow to extended family and community institutions, not just individual recipients; (Nelson and Gersbach, 2016) profit-sharing from HGGE-derived knowledge and research should fund community healthcare; (Caval and iere, 2018) governance structures should include community representatives, not just individual patients (Jecker, 2023). Hence, communal achievements should be both lineage and community, including people and institutions.

### 4.3 Public participation and deliberation in African biomedical governance

Public participation in HGGE governance is more than merely ethically imperative; evidence from African biomedical governance shows it strengthens policy legitimacy and identifies context-specific implementation barriers (Stilgoe et al., 2014). Notwithstanding, African case studies also reveal that declared participation divorced from resource investment in literacy, accessibility, and representation produces procedural inclusion masking substantive exclusion (Cletus Tandoh Andoh, 2017). We therefore ground our participation recommendations in empirical evidence of what enables authentic deliberation in African governance contexts.

Effective governance of emerging biomedical technologies in African contexts requires moving beyond technocratic models of scientific oversight toward deliberative frameworks grounded in procedural justice. We argue that three interrelated principles: inclusivity of relevant stakeholders*,* authenticity of deliberation*,* and institutional legitimacy, are imperative and essential for African HGGE governance.

The basis of inclusive stakeholder participation derives from democratic theory and relational justice. When biomedical governance affects populations bearing collective risks and potential benefits, procedural fairness requires that those affected have a meaningful voice in decision-making processes. Wanheng Hu demonstrated that public engagement frameworks embody symbolic imaginaries of “model citizens,” reflecting underlying assumptions about democratic participation (Hu, 2024). In African contexts, these assumptions must align with locally situated notions of democratic citizenship and communal relationships rather than replicating Western models. Research on scientist communication in South Africa further illustrates how institutional contexts, cultural backgrounds, and structural barriers shape scientists’ capacity to engage meaningfully with diverse publics (Magdelena Joubert Supervisor et al., 2018). Authentic deliberation requires more than consultation. Drawing from deliberative democracy theory, creating spaces where participants move beyond stated preferences to engage in informed, reciprocal reasoning with epistemic peers and experts is indeed a form of legitimate governance and participation (Hu, 2024). Institutional legitimacy requires that deliberative mechanisms be adequately funded and anchored in established bodies, such as Research Ethics Committees, rather than being constituted *ad hoc*. The H3Africa Consortium’s consultative workshops with RECs across African countries demonstrate a feasible operationalization model. Their structured forums allocated time for open discussion, supported by research funders, translating deliberative findings into governance guidance (Tindana et al., 2019).

These three principles are functionally essential rather than merely morally commendable. Public engagement increases trust in science, reduces subsequent controversy over governance decisions, and enables policies that reflect population values (Stilgoe et al., 2014; Burgess, 2014). For African HGGE governance, embedding procedural justice is thus both a matter of democratic principle and pragmatic governance design.

### 4.4 Why HGGE governance demands continental oversight

Effective governance of emerging technologies in heterogeneous contexts requires three structural features (Chan et al., 2015): polycentric authority distribution across governance tiers (Nelson and Gersbach, 2016), mechanisms for incorporating local knowledge and values into standard-setting, and (Caval and iere, 2018) adaptive review cycles to accommodate technological and social change (N and ikogosian, 2020). We examine how existing African regional governance bodies have operationalized these principles, and what HGGE governance can learn from their successes and limitations.

Polycentric authority distribution necessitates continental coordination. Evidence by Mwisongo and Nabyonga on global health initiatives in Africa demonstrates that when critical health technologies operate through parallel governance structures, separate funding channels, divergent reporting requirements, and fragmented coordination mechanisms, they overwhelm weak national capacity and create accountability gaps (Mwisongo and Nabyonga-Orem, 2016; Stephan et al., 2019). HGGE requires a baseline ground to build on, and these are deemed structural imperatives: research facilities, funding sources, and regulatory authorities. These have to be geographically dispersed across the continent and affixed to regional and global supporting bodies. Continental frameworks anchored in the African Union establish the institutional architecture through which national regulators, research ethics committees, and community bodies can coordinate without replicating parallel structures (Cubitt, 2014). This prevents what is referred to as regulatory arbitrage, which is when HGGE governance fragments across isolated national regimes; consequently, research migrates to jurisdictions with the weakest oversight. Polycentric authority, when effectively implemented, entails establishing a well-defined division of labor (Paniagua et al., 2021). Continental bodies set the minimum ethical standards and coordinate cross-border reviews, while national institutions incorporate these standards into their specific contexts.

Mechanisms for incorporating local knowledge and values demand continental staples. Individual African nations lack the analytical capacity and resources to independently negotiate with international HGGE developers, funders, and ethicists. Continental bodies enable pooling of bioethicists, geneticists, and community representatives across countries, creating deliberative capacity to articulate African philosophical frameworks, such as Ubuntu ethics, communal decision-making traditions, and health sovereignty, into binding governance standards and policies (Penfold and Fourie, 2015). Without this, HGGE standards reflect donor-defined models rather than African values. This complicates the overhauling process and reform efforts. Continental forums institutionalize authenticity and formalize resources for deliberation among diverse stakeholders across the continent, not *ad hoc* consultation after decisions are made (Gautier and Ridde, 2017).

Adaptive review cycles require African governance. HGGE is rapidly evolving technologically and socially. Emerging technology governance frameworks demonstrate that single national review bodies lack the comparative data and expertise to identify when standards require revision (Linkov et al., 2018). Continental institutions systematize monitoring of HGGE developments, coordinate periodic reassessment across nation-states, and adapt standards as evidence and societal expectations emerge, operationalizing institutional legitimacy through transparent, responsive processes that individual nations could fail to sustain alone (Gillard et al., 2017).

Hence, the case for continental oversight is structurally justified. For HGGE governance to establish effective polycentric authority, authentically incorporate African knowledge and values, or foster adaptive institutional learning, it will require a broader interlinkage beyond national-level arrangements alone.

### 4.5 The African HGGE governance framework: a continental harmonized model

A harmonized governance model is on the horizon. To achieve this, we propose a systematic, process-based framework that addresses the fragmentation in both continental and global systems. This framework will provide a mesh for actors to oversee, research, and practice germline gene editing. It is critical to name who makes decisions and by what authority. By doing so, we impose a responsibility and initiate accountability mechanisms that are responsive to these roles and characteristics. Therefore, institutional architecture is foundational. This means defined rules, standards, and binding instruments. It also means proper oversight through vigilant enforcement and proactive transparency. Eventually, inclusion of the society in decision-making bodies and institutions is crucial for the stability and continuity of such a model. On a deeper level, what is often overlooked are deliberation and evidence systems. These are situated within meaningful discourse, providing spaces for ethical questioning and moral inquiry. In other words, it’s a liberal discourse that’s community-based and places individuals, both present and future, at the forefront. Equally crucial is the procedural aspect of developing a mechanism that enables multi-stakeholder collaboration, ensuring their input is incorporated into the dialogue of HGGE. A base brick is the empirical component of evidence and data. To maintain and nurture the model, it’s crucial to encourage the scientific community to utilize and create research tools that perfectly gather public perspectives, concerns, and readiness. A functioning governance system necessitates rules, but only if they are enforced. Furthermore, meaningful community participation requires procedures. Unification must involve ethical deliberation, which will only be impactful if informed by data and evidence (World Health Organization, 2021).

#### 4.5.1 Foundational gap solution: establishing a continental regulatory framework

**FIGURE 2:**
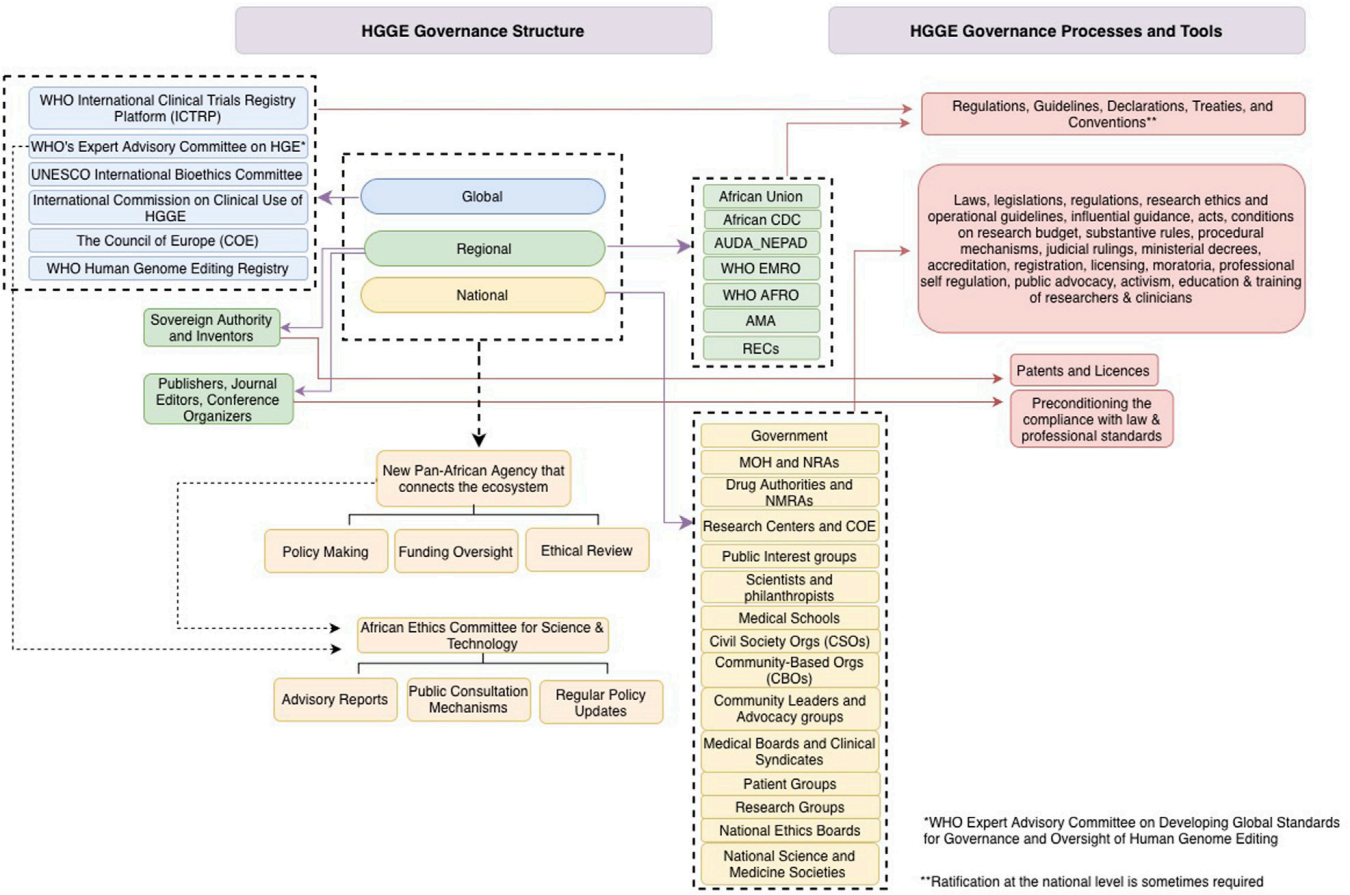
A conceptual framework for African HGGE governance framework (continental harmonized model).

**FIGURE 3:**
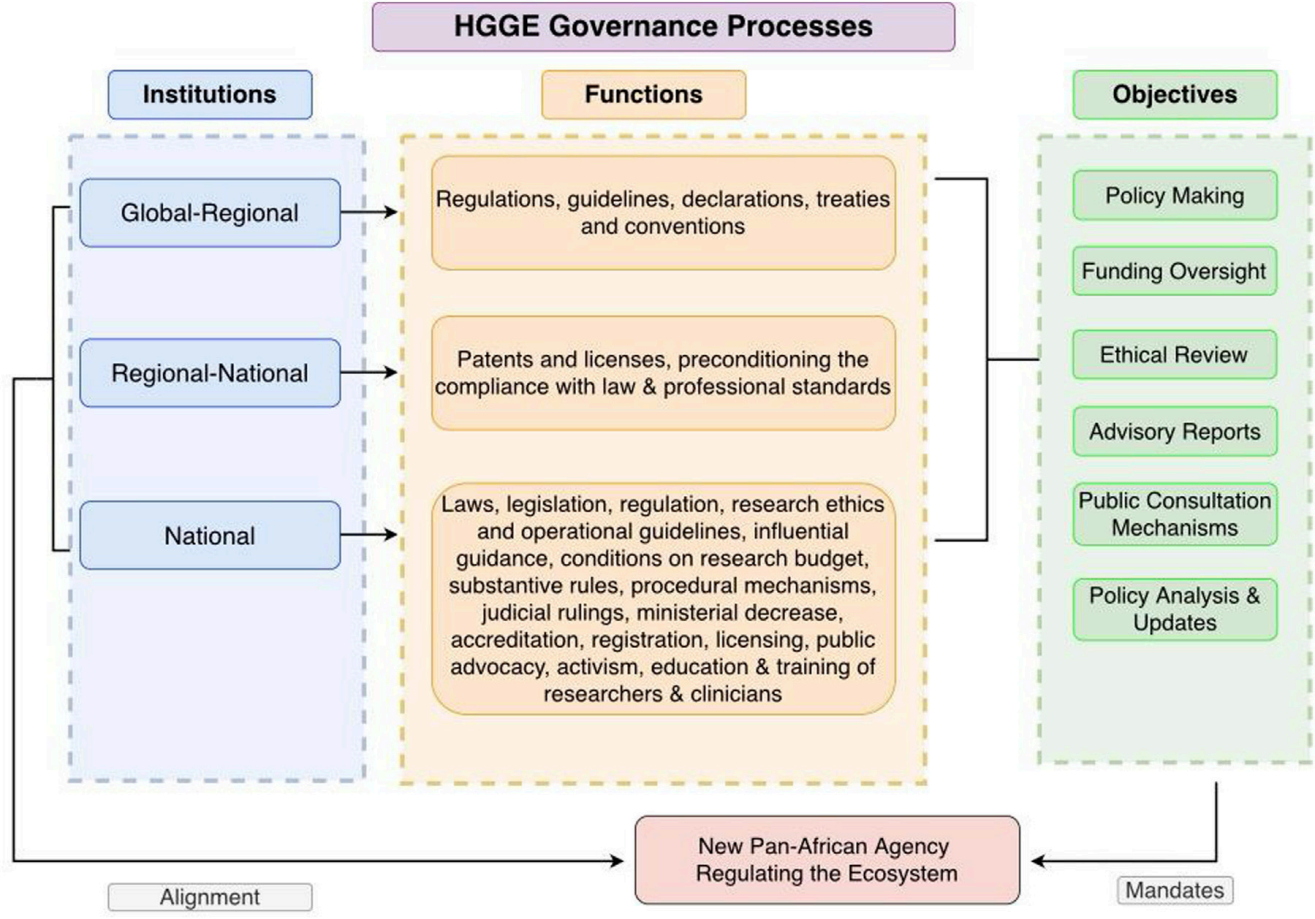
A conceptual framework for HGGE governance, institutional structure, and oversight actors in Africa.

**FIGURE 4:**
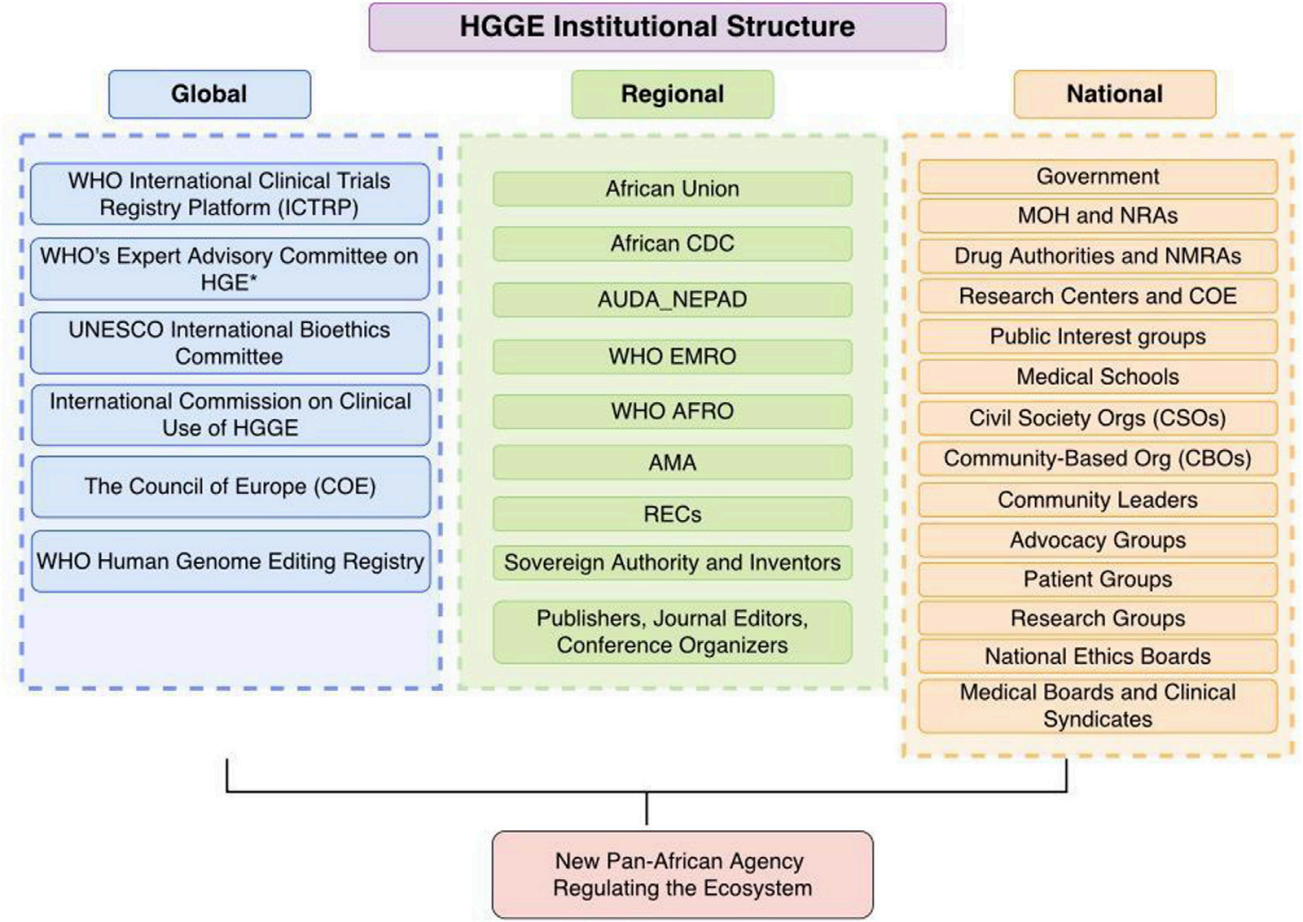
A conceptual framework for HGGE governance processes and oversight tools in Africa.

A plausible approach is to rely on the legislative efforts of the African nations; contrary to what Kaan et al. proposed, we strongly believe in a continental wave of action that could promote continental harmonization (Kaan et al., 2021). As a result, consistency and a sense of ownership would arise and harbor common objectives; consequently, a joint action and better access to futuristic technologies that tackle complex diseases and pathologies. In a concurrent pace with the continental regulatory development, the authors saw a huge opportunity for reforms in governance and policy action that should yield clear steps for adoption instead of negligence or abstention from using such a technology. Supported by resourceful international organizations, the AUDA-NEPAD health programme is now, along with the newly formed African Medicines Agency (AMA), going full power to harmonize the drug regulatory landscape in Africa, with most of the countries showing interest and willingness to benefit from this unprecedented unity in regulatory frameworks and medicines registration and access (African Union Development Agency Auda-nepad, 2025). As such, we envisaged the highly advanced tools used in HGGE as key health technology apparatuses that should be included in the game, channeling regulatory policies and approval schemes, more strict and more precise, but active and actionable. Building on Ubuntu philosophy and other available ethical foundations, along with communal values and standards, as mentioned in the previous sections, harmonization is the net outcome of this reform action. Embedding the African context inside the model as a core part is a normative foundation and consequently frames the entire argument of people-first ideology. HGGE governance in Africa would not commence in an institutional vacuum. A layered architecture of continental, regional, and national bodies already governs adjacent domains, and Figures 2–4 map how these layers interact. At the apex, the African Union (AU) holds the authority to adopt binding treaties and model legislation, but this authority is conditional rather than self-executing. The AMA’s Treaty, concluded in 2019 and entered into force in 2021, was drafted with a focus on regulating medicines, traditional medicines, and medical devices. The treaty’s negotiators have probably overlooked heritable genome editing; future individuals differ substantially from pharmaceutical safety and efficacy reviews. Extending AMA’s mandate to HGGE is therefore a normative proposal, not a description of existing legal fact. AU instruments bind only those member states that ratify and subsequently lead them, a structural limitation fully demonstrated by the African Medicines Agency (AMA) Treaty, which took longer than expected to reach its ratification threshold (Ncube et al., 2024).

#### 4.5.2 Oversight gap solution: developing integrated accountability and enforcement infrastructure

Therefore, reform should commence right from the roots, and accountability should be aligned with clear pathways for implementation and regulation. These measures are crucial in ensuring that future generations are not deprived of their rights or burdened with unchosen genetic flaws (World Health Organization, 2021). As a solution, we envision an African agency, either a new one or perhaps relying on existing agencies like AMA, for providing support in evaluating health technologies used in HGGE for AU-priority diseases and inspecting, coordinating, and sharing information on authorization across all member states. As shown in Figure 2, this new agency, in coordination with other local, regional, and international agencies, will lead joint clinical trial reviews, assess complex dossiers, and coordinate joint inspections of biotech companies and testing sites. By establishing common standards and regulations, as shown in Figure 4, this unified agency will drive regulatory harmonization across the continent. Working with Regional Economic Communities (RECs) and National Medicines Regulatory Authorities (NMRAs), ultimately, this will improve cross-country information sharing, as shown in Figure 3, which portrays the governing actors. National Medicines Regulatory Authorities (NMRAs) and Ministries of Health retain full sovereign licensing and inspection authority, but their technical maturity is highly uneven: as of late 2024, only a small number of African National Regulatory Authorities had reached WHO maturity level 3 of 4, with the remainder operating at lower capacity tiers (WHO, 2024). AMA would require a minimum of four conditions (Chan et al., 2015): legal clarification through Treaty amendment or an explicit AU Assembly or Conference of State Parties decision (Nelson and Gersbach, 2016); technical capacity beyond what AMA currently holds, given that the agency itself remains in the process of operationalizing its core medicines mandate years after its intended 2018 launch date; (Caval and iere, 2018) dedicated funding, distinct from existing AMRH and AMA budgets, to avoid diverting resources from medicines regulation; (Human Genome Editing, 2017) and member-state buy-in, a serious requirement, given that, and based on quite recent assessments, AMA ratification remains incomplete across the AU's full membership (Ncube et al., 2024). Presented this way, AMA is best understood as the most promising future host for HGGE oversight rather than its present regulator. This distinction should strengthen, rather than weaken, the manuscript’s normative argument by attuning institutional ambition to an established institutional reality.

#### 4.5.3 Societal gap solution: anchoring community representation in governance institutions

Involving the public is a fundamental step for proper deliberations, especially when the government starts to question what is right and what’s wrong ethically, socially, and religiously, leading to the adoption of a suitable direction or policy directly affecting the community. All of this is aimed towards socially aligned policy making, giving the full ground for all society groups, ranging from policymakers, clinicians, researchers, community leaders, and patient groups, ideally governing democratic rule of law and maintaining fundamental human rights (Kaan et al., 2021; Geuverink et al., 2024). Also, as mentioned before, various perspectives, with good representations typically from underrepresented society members, enrich dialogues and galvanize policies and laws. A proper assessment of the concepts of reducing disease burden, especially rare diseases, with means of gene editing, whether somatic or germline, is imminent for the successful uptake and generation of thoughts from society members. Hence, the responsibility falls on the leaders of these small-intermediate groups, along with continuous support from the ruling state (Martani, 2024). By drawing on evidence and research from various ethical and regulatory frameworks to study the approaches driving the current global policies on HGGE, the global biomedical ethics space needs local representation, which, as a result, should lead deliberations on regulations, societal inclusion, and ethical unification. They should all be rooted in the populations and concerned end-users, allowing transparent actions and permitting equitable reforms.

#### 4.5.4 Discursive gap solution: institutionalizing ethical deliberation

Blind spots in the discussion around HGGE will cease to exist once proper deliberations are established. The gaps and obstacles being faced while witnessing a promising clean-slate-like technology such as HGGE are quite tempting and could easily entice decision-makers and biotech giants to look over some of the delicate and sensitive consequences. Having an accessible space for ethical questioning rooted in African moral and religious traditions is essential for the continuity of the genome editing concept and its governance frame. Policymakers and clinicians need an institutional forum to deliberate these moral assumptions, which are usually left unexamined or unresolved. Evidence from Gillard et al. shows that this would not be feasible without national policies and local action from ministries of health and drug authorities, and even to form specialized agencies solely for conducting continuous ethical inquiry (Africa Centres for Disease Control and Prevention Africa CDC, 2025; Gillard et al., 2017). These entities are basically deliberative bodies that produce advisory opinions on the moral permissibility of specific HGGE applications, host structured dialogues on contested ethical dilemmas, and engage public moral reasoning on HGGE’s place in African minds. These institutions should be explicitly multi-faith and multicultural in composition, and their opinions should be published as public and accessible opinion pieces. This is what is called moral pluralism, where disagreement itself has been turned visible and transparent (Geuverink et al., 2024; Ndomondo-Sigonda et al., 2020).

#### 4.5.5 Procedural gap solution: designing stakeholder engagement mechanisms

AUDA-NEPAD, the AU’s technical and development-coordination agency, exercises convening and capacity-building authority rather than regulatory power. This convening role was precisely sustained over roughly a decade through the AMRH initiative, which gathered the political and technical consensus eventually codified in the AMA (Houtman et al., 2023). Nonetheless, readiness and acceptability should be foreseen beforehand as possible hindrances to such consensus and discussion (Ndomondo-Sigonda et al., 2020). The Africa CDC, the AU’s second specialized health agency, holds convening and technical guidance authority over public health emergencies and, increasingly, over continental genomic surveillance coordination. This is illustrated by its 2026 establishment of the African Strategic Advisory Group on Genomics, a body explicitly tasked with advising on data governance, ethics, and benefit-sharing (Africa CDC, 2026). However, this authority does not extend to clinical research ethics or genome-editing oversight specifically, which we propose to be left for the new agency to manage. WHO’s regional offices (AFRO and EMRO) supply technical guidance and link national systems to global instruments such as the WHO Human Genome Editing Registry, but, consistent with WHO’s constitution, they work collaboratively rather than compel adoption, leaving sovereignty over health regulation with member states (WHO, 2019). Regional Economic Communities (RECs) such as the East African Community have hosted the continent’s most advanced harmonization pilots, including a joint dossier-assessment procedure that measurably shortened regulatory timelines over its first 8 years of operation, yet REC authority binds only its own membership, and enrollment remains uneven across the continent (Mashingia et al., 2020). Among this architecture, AUDA-NEPAD and AMA constitute the most institutionally efficient anchors for a future HGGE oversight function, precisely because they already possess what no purpose-built HGGE institution yet has: a harmonized continental review infrastructure, treaty-based legal personality, and working operational relationships with NMRAs across the continent. This relevance, however, has firm and legally consequential limits that the manuscript must state explicitly rather than imply.

#### 4.5.6 Empirical gap solution: building public perspectives databases

A fundamental issue to align HGGE governance in African societies is the systematic data on how different populations, including those burdened with rare diseases, families carrying genetic conditions, clinicians, researchers, and lay persons, understand and respond to germline gene editing as a healthcare intervention (Kaan et al., 2021; African Union Development Agency Auda-Nepad, 2025). To avoid this, policy discussions should have empirical grounding, especially since Africa has an inherited variety and complexity in the social mesh. It harbors diverse religious traditions, family structures, and different understandings of genetic risk and disease prevention, along with a historical lack of genetic counseling and literacy. Various African countries have made strides in the genomics field, leading their national genome projects. These triumphs are cataloged as hope for HGGE adoption and access (Africa Centres for Disease Control and Prevention Africa CDC, 2025). With eight running projects and multiple multi-country initiatives, this will ensure proper governance and a huge influx of data that consequently will accumulate efforts and generate political will for a future less burdened by complex and rare diseases, as shown in Table 1. African genome projects and national genomics initiatives offer institutional platforms for embedding public perspectives, surveys and longitudinal monitoring of continuously evolving knowledge and attitudes toward gene editing. The resulting databases should capture public opinion in a granular, stratified way. Perspectives should be aggregated by disease profile: rare, common, and severe. Also, geographic region, religion, cultural background, and gender. Consequently, a true feedback mechanism will be established, delivering targeted education and stimulate ethical deliberation before even policies are set (Kaan et al., 2021; Geuverink et al., 2024). The operational structure of public perspectives databases and knowledge assets should be integrated into the layered governance architecture proposed above. National ethics committee could design context-specific survey instruments. Continental and regional coordinating bodies should assist in harmonizing data collection. A continental registry will house anonymized, searchable public-data components linked to clinical and research registries. As part of data governance solid protocols should ensure that public perspectives database embody equity principles. The collected data should remain accessible to African researchers and policymakers. Data should be disaggregated to avoid erasing demographic heterogeneity. Periodic reporting of findings should feed directly into policy revision cycles. The reports should focus solely on attitudes, concerns, and evolving consensus of the African populations (Ndomondo-Sigonda et al., 2020). Hence, the outcome is two things: first, regulatory decisions will be grounded on documented public priorities, second, this will tell whether interventions education, community forums, and pilot clinical programs are shifting understanding and reducing barriers to equitable HGGE access or not (Martani, 2024).

**TABLE 1:** Genome projects across Africa.

Country	Name of the project	Details
South Africa	The Southern African Human Genome Programme (SAHGP)	The programme launched back in 2011 as the oldest in the continent, and aiming to scale up to a large 110 thousand cohort project (European Genome-phenome Archive EGA, 2025)
Egypt	The Egypt Genome Project	The project revealed initial reports in 2024, and just recently published full results of more than one thousand Egyptians, showcasing novel population characteristics and announcing a national triumph in genomics. Egypt also showed a high level of leadership in May 2025 in Geneva by sponsoring the resolution entitled “Rare Diseases: A priority for Global Health Equity and Inclusion” at the World Health Assembly (WHA) 78 (Elmonem et al., 2024; Amer et al., 2026)
Morocco	Moroccan Genome Project (MGP)	Morocco has published Phase-1 results sequencing 109 Moroccan genomes in 2025 (El Fahime et al., 2025).
Nigeria	The Nigerian 100 thousand Genome Project	The project is led by 54gene and the African Centre for Translational Genetics, completing its first phase announced in 2022 (Fatumo et al., 2022a)
Ghana	GhGenome-1000 Ghanaian Genomes	The project is led by the West African Genetic Medicine Centre (WAGMC) launched in 2022 (West African Genetic Medicine Centre WAGMC, 2025)
Uganda	Uganda Genome Resource (UGR)	It is not a national genome but rather a large resource used in population genetics research, both genotype and WGS data (Fatumo et al., 2022b)
The Gambia	Gambian Genome variation Project (GGVP)	It is not quite a national genome project but rather led by the MRC Unit which’s a national data bank since 2000 (Vaught, 2017)
Kenya	Turkana Genomic Project	It is not a real genome project, but it studies a Kenyan tribe, Turkana tribe, and generally Kenya hosts KEMRI and other partners operating genomics research that includes NGS, sequencing and bioinformatics studies (Khemchandani and Taylor, 2022; Mpala Research Center)
Multiple Countries	H3Africa, the African Genome Variation Project, the African BioGenome Project, and Roche’s African Genomics Program	They are all considered Pan-African and multi-country initiatives that coordinate funding for biorepositories, mainly in Nigeria, Uganda, and South Africa, and continental projects, facilities networking programs, and lead capacity building programs (African BioGenome Project, 2025; Wonkam, 2021; Gurdasani et al., 2015; Roche, 2025)

## 5 Policy recommendations

### 5.1 National foundation

- Define clear agency roles. For instance, and not exclusively, the Ministry of Health for clinical oversight, the National Science and Technology Authority for research funding, and the national drug regulator for gene therapy products regulation. This could avoid overlapping responsibilities (Zou et al., 2025).
- Pass a forward-looking, adaptable legislation that updates biosafety, genetic resource, and ethical review regulations every defined number of years (Zou et al., 2025).
- Establish a National Ethics Committee for Genome Editing with a mandate to review proposals, monitor long-term outcomes, and publish annual accountability reports (Zou et al., 2025).
- Institutionalize stakeholder dialogue: involve patient groups, NGOs, the media, and the public through regular consultations and transparent platforms (Magdelena Joubert Supervisor et al., 2018).
- Mandate bioethics education and training for scientists, clinicians, and regulators (Munung et al., 2024).

### 5.2 Continental coordination

- Create a pan-African coordinating body (under the AU Health Commission or hosted by AMA) that:
o Establishes a unified African HGGE Framework that classifies genome-editing activities (basic research, preclinical, clinical, and heritable)
o Assigns lead agencies, e.g., Africa CDC, for surveillance
o Issues continent-wide HGGE guidelines harmonizing member-state legislation
- Establish an African Ethics Committee for Science and Technology to provide advisory reports, public consultation mechanisms, and regular policy updates (Zou et al., 2025).
- Establish continental data governance standards, balancing protection with collaborative access (Ssebunnya, 2023).
- Establish hybrid consent and access committee models (Rebai et al., 2025).
- Clarify benefit sharing, data custodianship, and controlled access protocols to maintain anonymization while enabling transparent collaboration (Kabata and Thaldar, 2023; Rebai et al., 2025; Thaldar, 2024; Munun et al., 2025).
- Launch voluntary public engagement initiatives.
- Scale trust-building and consent methodologies across member states.
- Institutionalize evidence-based participation models.

### 5.3 Operationalization

- Secure AU and member states’ ratification of expanded AMA Treaty authority over HGGE (contingent on Phase 2 success) (Ncube et al., 2024; Mashingia et al., 2020).
- Formalize the continental HGGE Framework as binding legislation.
- Establish cross-border regulatory cooperation: joint clinical trial reviews, coordinated dossier assessment, and harmonized inspections (Penfold and Fourie, 2015).
- Establish a continental HGGE registry linked to the WHO Human Genome Editing Registry and the WHO International Clinical Trials Registry Platform (WHO, 2019).
- Implement a cross-country information-sharing infrastructure for real-time authorization tracking (Thaldar, 2024).
- Create joint inspection and oversight mechanisms coordinating NMRAs and regional economic communities (RECs) (Rebai et al., 2025).
- Leverage the WHO, country, and regional offices (AFRO and EMRO), and other international advisory panels for technical guidance and capacity-building (World Health Organization, 2021).
- Embed HGGE oversight within existing ethics committees and NMRAs (risk mitigation: to avoid the prospect of creating redundant, poorly funded agencies) (Eric Green, 2022).
- Experts warn of superficial, low-literacy consultation, addressed by reusing this tested methodology rather than bringing out more innovative processes (Munung et al., 2024; Eric Green, 2022).
- Operationalize tested H3Africa protocols and ELSI projects and avoid inventing new mechanisms (keeping in mind this counterargument: Experts warn of superficial, low-literacy consultation, addressed by reusing this tested methodology rather than bringing out more innovative processes) (Munung et al., 2024; Eric Green, 2022).
- Maintain African institutional leadership while embedding global standards (Munung et al., 2024; Eric Green, 2022)

## 6 Feasibility and phased implementation roadmap

**FIGURE 5:**
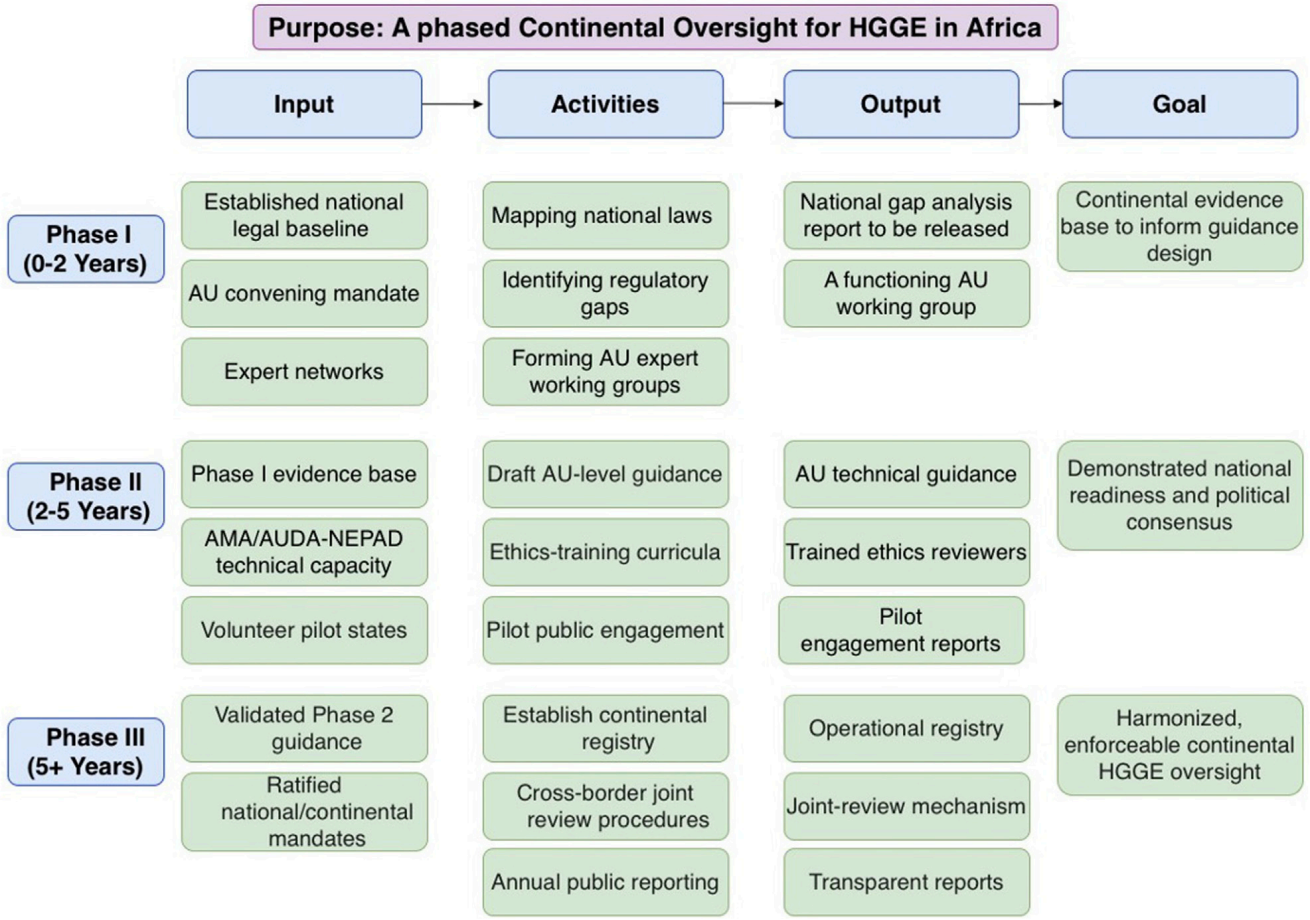
A logic model of the phased HGGE governance roadmap: input, activities, output, and goals for each implementation phase, nested under the overarching purpose.

Continental harmonization is best understood as a multi-stage institutional process rather than a single legislative event as shown in Figure 5, governed by three principles (Chan et al., 2015): subsidiarity, where authority rests at a lower competent level until readiness is demonstrated (Nelson and Gersbach, 2016); gradual trust-building, where a formal mandate tracks demonstrated capacity; and (Caval and iere, 2018) precautionary balance, whereby oversight intensifies as research approaches clinical translation. This sequencing mirrors the African Medicines Regulatory Harmonization (AMRH) trajectory, in which roughly a decade of regional convergence preceded the African Medicines Agency (AMA) Treaty, and ratification itself required several additional years to reach effect. A shorter timeline risks granting formal authority before national legal and technical capacity exists, which the literature identifies as the principal cause of hindered implementation in comparable African regulatory harmonization efforts (Ncube et al., 2024; Houtman et al., 2023).

The continent’s recent experience with pathogen genomic surveillance demonstrates that pan-African scientific coordination can scale quickly once political will and minimal infrastructure align, illustrated by the rapid expansion of sequencing capacity during the COVID-19 response.

Realistically, three risks could still derail the timeline, and each has a concrete mitigation already embedded in the roadmap design. First, financing gaps, which historically slowed AMA’s own ratification, can be partly addressed by anchoring phase I mapping work within existing AUDA-NEPAD and Africa CDC budget lines rather than requiring new appropriations (Ncube et al., 2024). Second, uneven national regulatory maturity, evident in the fact that only a small minority of African National Regulatory Authorities currently reach WHO maturity level 3, is mitigated by sequencing pilot engagement in phase II toward the more capable jurisdictions first, allowing lessons to transfer outward rather than requiring uniform readiness at the outset (WHO, 2024). Third, political fragmentation across Regional Economic Communities can be mitigated by deliberately piloting phase II guidance within a small number of willing RECs before seeking AU Assembly-wide endorsement in Phase III, an approach that already proved workable for medicines harmonization through the East African Community’s joint-assessment mechanism (Mashingia et al., 2020).

## 7 Conclusion

In conclusion, the HGGE technology application in research and practice is a critical dilemma in modern medicine. It poses societal, ethical, and political issues not only on the national level but globally as well. Many ethical principles are highly relevant to the use of HGGE; the concepts of beneficence and nonmaleficence reveal the moral duty to mitigate suffering from serious hereditary diseases and the mandate to avoid irreversible harm, especially within the huge uncertainty that exists in the scientific field. Similarly, access to HGGE exclusively by the wealthy population who can afford the high cost raises concerns under the ethical principle of justice. Questions arise regarding the autonomy of future individuals who are unable to consent to genetic alterations that alter their identity. African ethics adopts a more mutual philosophy in comparison to the individualist-based Western bioethical values. Thus, relying on continental and local knowledge, values, and capabilities to develop the best practices on how to oversee them is essential. Currently, the landscape of global as well as the continental governance is highly fragmented and inconsistent, paving the way for failure in addressing foundational, oversight, and societal gaps. Particularly, in the absence of comprehensive public engagement, the WHO must be a fundamental stakeholder in the HGGE application, supported by the various regional actors. If HGGE is ever to be integrated in modern medicine, it must be implemented under a robust global governance mechanism that addresses transparency, accountability, standardized science and definition, and equity. The continent’s biotechnological maturity is evident in its various genome projects and connected communities. These promoters, along with others, can accelerate the adoption, development, and localization of such technologies, opening the door for more lifesaving and life-changing interventions. Good governance begins at the grassroots level, allowing full inclusion and involvement, not just participation. Cross-border participation, innovation, and funding will maximize benefits while putting ethical practice in the forefront. Therefore, not only will ethical progress depend solely on advancements in technology, but also on humanity`s collective capacity to govern innovation with humility, equity, and foresight.

## References

[B1] Africa CDC (2026). Africa CDC’s strategic advisory group on genomics. Available online at: https://africacdc.org/news-item/africa-cdc-launches-the-african-strategic-advisory-group-on-genomics/ (Accessed June 22, 2026).

[B2] Africa Centres for Disease Control and Prevention (Africa CDC) (2025). Launch of Africa genome archiving for response and insight (AGARI). Available online at: https://africacdc.org/news-item/africa-cdc-launches-agari-a-continent-wide-genomic-data-platform-to-strengthen-outbreak-response/ (Accessed December 1, 2025).

[B3] African BioGenome Project (2025). African BioGenome project. Available online at: https://africanbiogenome.org (Accessed December 14, 2025).

[B4] African Union Development Agency (AUDA-NEPAD) (2025). African medicines agency (AMA). Available online at: https://www.nepad.org/microsite/african-medicines-agency-ama (Accessed November 11, 2025).

[B5] Almeida M. Ranisch R. (2022). Beyond safety: mapping the ethical debate on heritable genome editing interventions. Humanit Soc. Sci. Commun. 9 (1), 139. 10.1057/s41599-022-01147-y

[B6] Alonso M. Savulescu J. (2021). He Jiankui´s gene‐editing experiment and the non‐identity problem. Bioethics 35 (6), 563–573. 10.1111/bioe.12878 33951203 PMC8524470

[B7] Amer K. Moustafa A. Hassan W. A. Adel E. AbdElaal K. R. Ghanim T. A. (2026). EGP1K: whole-genome sequencing of 1,024 egyptians characterizes population structure and genetic diversity. bioRxiv. 10.64898/2026.04.02.715521

[B8] Andorno R. Baylis F. Darnovsky M. Dickenson D. Haker H. Hasson K. (2020). Geneva statement on heritable human genome editing: the need for course correction. Trends Biotechnol. 38 (4), 351–354. 10.1016/j.tibtech.2019.12.022 32014274

[B9] ANYE-NKWENTI NYAMNJOH (2025). Shaping the future of technology ethics from Africa. Available online at: https://health.uct.ac.za/ethics-lab/articles/2025-12-05-shaping-future-technology-ethics-africa (Accessed June 15, 2026).

[B10] Baumann M. (2016). CRISPR/Cas9 genome editing – new and old ethical issues arising from a revolutionary technology. Nanoethics 10 (2), 139–159. 10.1007/s11569-016-0259-0

[B11] Baylis F. (2019). Altered Inheritance : CRISPR and the Ethics of Human Genome Editing. Harvard University Press. Available online at: https://www.hup.harvard.edu/books/9780674976719 (Accessed November 6, 2025).

[B12] Baylis F. Darnovsky M. Hasson K. Krahn T. M. (2020). Human germline and heritable genome editing: the global policy landscape. CRISPR J. 3 (5), 365–377. 10.1089/crispr.2020.0082 33095042

[B13] Brokowski C. Adli M. (2019). CRISPR ethics: moral considerations for applications of a powerful tool. J. Mol. Biol. 431 (1), 88–101. 10.1016/j.jmb.2018.05.044 29885329 PMC6286228

[B14] Burgess M. M. (2014). From ‘trust us’ to participatory governance: deliberative publics and science policy. Public Underst. Sci. 23 (1), 48–52. 10.1177/0963662512472160 24434712

[B15] Cavaliere G. (2018). Genome editing and assisted reproduction: curing embryos, society or prospective parents? Med. Health Care Philos. 21 (2), 215–225. 10.1007/s11019-017-9793-y 28725950 PMC5956052

[B16] Cavaliere G. Devolder K. Giubilini A. (2019). Regulating genome editing: for an enlightened democratic governance. Camb. Q. Healthc. Ethics 28 (1), 76–88. 10.1017/S0963180118000403 30570466 PMC6316359

[B17] Chan S. Donovan P. J. Douglas T. Gyngell C. Harris J. Lovell-Badge R. (2015). Genome editing technologies and human germline genetic modification: the hinxton group consensus statement. Am. J. Bioeth. 15 (12), 42–47. 10.1080/15265161.2015.1103814 26632362

[B18] Cletus Tandoh Andoh (2017). Genome editing technologies: ethical and regulation challenges for Africa. Int. J. Health Econ. Policy 2 (2), 30–46. 10.11648/j.hep.20170202.11

[B19] Coller B. S. (2019). Ethics of human genome editing. Annu. Rev. Med. 70 (1), 289–305. 10.1146/annurev-med-112717-094629 30691366 PMC11299715

[B20] Conley J. M. Cadigan R. J. Davis A. M. Juengst E. T. Kuczynski K. Major R. (2023). The promise and reality of public engagement in the governance of human genome editing research. Am. J. Bioeth. 23 (7), 9–16. 10.1080/15265161.2023.2207502 PMC10367578 37204137

[B21] Council of Europe (COE) (1997). Convention for the protection of human rights and dignity of the human being with regard to the application of biology and medicine: convention on human rights and biomedicine (ETS no. 164) (oviedo). Available online at: https://www.coe.int/en/web/human-rights-and-biomedicine/oviedo-convention (Accessed November 2, 2025).

[B22] Cubitt C. (2014). An introduction to governance in Africa. Gov. Afr. 1 (1), 1. 10.5334/gia.ae

[B23] Cyranoski D. Ledford H. (2018). Genome-edited baby claim provokes international outcry. Nature 563 (7733), 607–608. 10.1038/d41586-018-07545-0 30482929

[B24] Deplazes-Zemp A. (2019). Challenges of justice in the context of plant genetic resources. Front. Plant Sci. 10, 1266. 10.3389/fpls.2019.01266 31708939 PMC6823224

[B25] El Fahime E. Kartti S. Chemao-Elfihri M. W. Festali R. Hakmi M. Ibrahimi A. (2025). Moroccan genome project: genomic insight into a North African population. Commun. Biol. 8 (1), 584. 10.1038/s42003-025-08020-z 40204857 PMC11982406

[B26] Elmonem M. A. Soliman N. A. Moustafa A. Gad Y. Z. Hassan W. A. Taha T. (2024). The Egypt genome project. Nat. Genet. 56 (6), 1035–1037. 10.1038/s41588-024-01739-1 38684896

[B27] Eric Green M. D. (2022). H3Africa program comes to an end, leaving an important genomics legacy. Available online at: https://www.genome.gov/about-nhgri/Director/genomics-landscape/nov-3-2022-h3africa-program-comes-to-an-end-leaving-an-important-genomics-legacy (Accessed June 22, 2026).

[B28] European Genome-phenome Archive (EGA) (2025). The southern African human genome programme. Available online at: https://ega-archive.org/studies/EGAS00001002639 (Accessed December 12, 2025).

[B29] Evans J. H. (2021). Setting ethical limits on human gene editing after the fall of the somatic/germline barrier. Proc. Natl. Acad. Sci. 118 (22), e2004837117. 10.1073/pnas.2004837117 34050016 PMC8179225

[B30] Ewuoso C. Wonkam A. de Vries J. (2022). Epistemic justice, African values and feedback of findings in African genomics research. Glob. Bioeth. 33 (1), 122–132. 10.1080/11287462.2022.2124019 36185769 PMC9518233

[B31] Fatumo S. Yakubu A. Oyedele O. Popoola J. Attipoe D. A. Eze-Echesi G. (2022a). Promoting the genomic revolution in Africa through the Nigerian 100K genome project. Nat. Genet. 54 (5), 531–536. 10.1038/s41588-022-01071-6 35534563

[B32] Fatumo S. Mugisha J. Soremekun O. S. Kalungi A. Mayanja R. Kintu C. (2022b). Uganda genome resource: a rich research database for genomic studies of communicable and non-communicable diseases in Africa. Cell Genomics 2 (11), 100209. 10.1016/j.xgen.2022.100209 36388767 PMC9646479

[B33] Faure M. C. Munung N. S. Ntusi N. A. B. Pratt B. de Vries J. (2021). Considering equity in global health collaborations: a qualitative study on experiences of equity. PLoS One 16 (10), e0258286. 10.1371/journal.pone.0258286 34618864 PMC8496851

[B34] Gautier L. Ridde V. (2017). Health financing policies in Sub-Saharan Africa: government ownership or donors’ influence? A scoping review of policymaking processes. Glob. Health Res. Policy 2 (1), 23. 10.1186/s41256-017-0043-x 29202091 PMC5683243

[B35] Genetic Literacy Project (2020). Global gene editing regulation tracker and index. Available online at: https://crispr-gene-editing-regs-tracker.geneticliteracyproject.org/ (Accessed November 9, 2025).

[B36] Geuverink W. P. Houtman D. Retel Helmrich I. R. A. van Baalen S. van Beers B. C. van El C. G. (2024). The need to set explicit goals for human germline gene editing public dialogues. J. Community Genet. 15 (3), 259–265. 10.1007/s12687-024-00710-1 38720104 PMC11217238

[B37] Gillard R. Gouldson A. Paavola J. Van Alstine J. (2017). Can national policy blockages accelerate the development of polycentric governance? Evidence from climate change policy in the United Kingdom. Glob. Environ. Change 45, 174–182. 10.1016/j.gloenvcha.2017.06.003

[B38] Greely H. T. (2019). CRISPR’d babies: human germline genome editing in the ‘he jiankui affair. J. Law Biosci. 6 (1), 111–183. 10.1093/jlb/lsz010 31666967 PMC6813942

[B39] Gurdasani D. Carstensen T. Tekola-Ayele F. Pagani L. Tachmazidou I. Hatzikotoulas K. (2015). The African genome variation project shapes medical genetics in Africa. Nature 517 (7534), 327–332. 10.1038/nature13997 25470054 PMC4297536

[B40] Gyngell C. Douglas T. Savulescu J. (2017). The ethics of germline gene editing. J. Appl. Philos. 34 (4), 498–513. 10.1111/japp.12249 28919655 PMC5573992

[B41] Habermas J. (2003). The Future of Human Nature. Cambridge, United Kingdom: Polity Press. Available online at: https://monoskop.org/images/3/36/Habermas_Jürgen_The_Future_of_Human_Nature_2003.pdf (Accessed November 5, 2025).

[B42] Halley M. C. Halverson C. M. E. Tabor H. K. Goldenberg A. J. (2023). Rare disease, advocacy and justice: intersecting disparities in research and clinical care. Am. J. Bioeth. 23 (7), 17–26. 10.1080/15265161.2023.2207500 PMC10321139 37204146

[B43] Heritable Human Genome Editing (2020). Washington, D.C.: National Academies Press. 10.17226/25665 32897669

[B44] Houtman D. Geuverink W. Helmrich IRAR Vijlbrief B. Cornel M. Riedijk S. (2023). “What if” should precede “whether” and “how” in the social conversation around human germline gene editing. J. Community Genet. 14 (4), 371–375. 10.1007/s12687-023-00652-0 37326787 PMC10444910

[B45] Hu W. (2024). Imagining the model citizen: a comparison between public understanding of science, public engagement in science, and citizen science. Public Underst. Sci. 33 (6), 709–724. 10.1177/09636625241227081 38369701

[B46] Human Genome Editing. (2017). Washington, D.C.: National Academies Press; 10.17226/24623

[B47] Hyder A. A. Ali J. Hallez K. White T. Sewankambo N. K. Kass N. E. (2015). Exploring institutional research ethics systems: a case study from Uganda. AJOB Empir. Bioeth. 6 (3), 1–14. 10.1080/23294515.2014.981316 26594648 PMC4652948

[B48] Iltis A. S. Hoover S. Matthews K. R. W. (2021). Public and stakeholder engagement in developing human heritable genome editing policies: what does it mean and what should it mean? Front. Polit. Sci. 3, 730869. 10.3389/fpos.2021.730869

[B49] Imafidon E. (2022). “African ethno-ethics and bioethical principlism: implication for the othered patient,” in Ethnophilosophy and the Search for the Wellspring of African Philosophy (Cham: Springer International Publishing), 175–187. 10.1007/978-3-030-78897-1_11

[B50] International Bioethics Committee (IBC) (2021). Report of the international bioethics committee (IBC) on the principle of protecting future generations. Available online at: https://unesdoc.unesco.org/ark:/48223/pf0000378723 (Accessed November 8, 2025).

[B51] Jasanoff S. Hurlbut J. B. Saha K. (2019). Democratic governance of human germline genome editing. CRISPR J. 2 (5), 266–271. 10.1089/crispr.2019.0047 31599682 PMC7476380

[B52] Jecker N. S. (2023). Ubuntu and bioethics. 161–180. 10.1007/978-3-031-25149-8_6

[B53] Joseph A. M. Karas M. Ramadan Y. Joubran E. Jacobs R. J. (2022). Ethical perspectives of therapeutic human genome editing from multiple and diverse viewpoints: a scoping review. Cureus. 14. 10.7759/cureus.31927 PMC9793437 36582559

[B54] Kaan T. Xafis V. Schaefer G. O. Zhu Y. Labude M. K. Chadwick R. (2021). Germline genome editing: Moratorium, hard law, or an informed adaptive consensus? PLoS Genet. 17 (9), e1009742. 10.1371/journal.pgen.1009742 34499642 PMC8428541

[B55] Kabata F. Thaldar D. (2023). Regulating human genomic research in Africa: why a human rights approach is a more promising conceptual framework than genomic sovereignty. Front. Genet. 30, 14. 10.3389/fgene.2023.1208606 PMC10347388 37456664

[B56] Kannan S. Najjar D. (2020). Therapeutic gene editing is here, can regulations keep up? MIT Sci. Policy Rev. 1, 63–69. 10.38105/spr.czm9c2w8ig

[B57] Kevles D. J. (2009). Eugenics, the genome, and human rights. Med. Stud. 1 (2), 85–93. 10.1007/s12376-009-0010-z

[B58] Khemchandani A. Taylor C. (2022). World of genomics: kenya. Front line genomics. Available online at: https://frontlinegenomics.com/world-of-genomics-kenya/ (Accessed December 13, 2025).

[B59] Kobayashi S. Miyoshi T. Kobayashi T. Hayakawa I. Urayama K. Y. Uchiyama M. (2022). Public attitudes in the clinical application of genome editing on human embryos in Japan: a cross-sectional survey across multiple stakeholders. J. Hum. Genet. 67 (9), 541–546. 10.1038/s10038-022-01042-z 35534678

[B60] Lander E. S. Baylis F. Zhang F. Charpentier E. Berg P. Bourgain C. (2019). Adopt a moratorium on heritable genome editing. Nature 567 (7747), 165–168. 10.1038/d41586-019-00726-5 30867611

[B61] Lanphier E. Urnov F. Haecker S. E. Werner M. Smolenski J. (2015). Don’t edit the human germ line. Nature 519 (7544), 410–411. 10.1038/519410a 25810189

[B62] Lee T. L. Sawai T. (2024). Navigating equity in global access to genome therapy expanding access to potentially transformative therapies and benefiting those in need requires global policy changes. Front. Genet. 4, 15. 10.3389/fgene.2024.1381172 PMC11024294 38638119

[B63] Linkov I. Trump B. D. Anklam E. Berube D. Boisseasu P. Cummings C. (2018). Comparative, collaborative, and integrative risk governance for emerging technologies. Environ. Syst. Decis. 38 (2), 170–176. 10.1007/s10669-018-9686-5 37829286 PMC10569133

[B64] Magdelena Joubert Supervisor C. Peter Weingart P. Johann Mouton P. (2018). Factors influencing the public communication behaviour of publicly visible scientists in South Africa. Available online at: https://scholar.sun.ac.za (Accessed May 7, 2026).

[B65] Major R. M. Davis A. M. Henderson G. E. Inamine G. Conley J. M. (2024). The public-private research ecosystem in the genome editing era. iScience 27 (6), 109896. 10.1016/j.isci.2024.109896 38784021 PMC11112366

[B66] Martani A. (2024). Changing the regulation of human germline genome editing: what does a truly broad societal debate entail? Law Innov. Technol. 16 (2), 687–714. 10.1080/17579961.2024.2392929

[B67] Mashingia J. H. Ahonkhai V. Aineplan N. Ambali A. Angole A. Arik M. (2020). Eight years of the east African community medicines regulatory harmonization initiative: implementation, progress, and lessons learned. PLoS Med. 17 (8), e1003134. 10.1371/journal.pmed.1003134 32785219 PMC7423058

[B68] McCaughey T. Sanfilippo P. G. Gooden G. E. C. Budden D. M. Fan L. Fenwick E. (2016). A global social media survey of attitudes to human genome editing. Cell Stem Cell 18 (5), 569–572. 10.1016/j.stem.2016.04.011 27152441

[B69] Mpala Research Center Turkana genomics project. Available online at: https://mpala.org/turkana-genomics-project/ (Accessed December 20, 2025).

[B70] Mulvihill J. J. Capps B. Joly Y. Lysaght T. Zwart H. A. E. Chadwick R. (2017). Ethical issues of CRISPR technology and gene editing through the lens of solidarity. Br. Med. Bull. 122 (1), 17–29. 10.1093/bmb/ldx002 28334154

[B71] Munung N. S. Ewuoso C. Chimusa E. R. Ogundiran T. (2025). Cultivating an equity-oriented data sharing culture for African health research initiatives. Nat. Commun. 16 (1), 8122. 10.1038/s41467-025-63289-2 40885725 PMC12398551

[B72] Munung N. S. de Vries J. Pratt B. (2022). Towards equitable genomics governance in Africa: guiding principles from theories of global health governance and the African moral theory of ubuntu. Bioethics 36 (4), 411–422. 10.1111/bioe.12995 35041227 PMC9050794

[B73] Munung N. S. Royal C. D. de Kock C. Awandare G. Nembaware V. Nguefack S. (2024). Genomics and health data governance in Africa: democratize the use of big data and popularize public engagement. Hastings Cent. Rep. 54 (S2). 10.1002/hast.4933 39707935

[B74] Mwisongo A. Nabyonga-Orem J. (2016). Global health initiatives in Africa – governance, priorities, harmonisation and alignment. BMC Health Serv. Res. 16 (S4), 212. 10.1186/s12913-016-1448-9 27454542 PMC4959383

[B75] Nikogosian H. (2020). Regional integration, health policy and global health. Glob. Policy 11 (4), 508–514. 10.1111/1758-5899.12835

[B76] Ncube B. M. Dube A. Ward K. (2024). The process of ratifying the treaty to establish the African medicines agency: perspectives of national regulatory agencies. Health Policy Plan. 39 (5), 447–456. 10.1093/heapol/czae017 38497780 PMC11095264

[B77] Ndomondo-Sigonda M. Mahlangu G. Agama-Anyetei M. Cooke E. (2020). A new approach to an old problem: overview of the east African Community’s medicines regulatory harmonization initiative. PLoS Med. 17 (8), e1003099. 10.1371/journal.pmed.1003099 32785223 PMC7423057

[B78] Nelson C. E. Gersbach C. A. (2016). Engineering delivery vehicles for genome editing. Annu. Rev. Chem. Biomol. Eng. 7 (1), 637–662. 10.1146/annurev-chembioeng-080615-034711 27146557

[B79] Nuffield Council on Bioethics (2018). Genome editing and human reproduction: social and ethical issues. Available online at: https://www.nuffieldbioethics.org/publication/genome-editing-and-human-reproduction-social-and-ethical-issues/ (Accessed November 5, 2025).

[B80] Paniagua P. Thiel A. Blomquist W. A. Garrick E. A. (2021). “Governing complexity: analyzing and applying polycentricity. Public Choice 188, 577–581. 10.1007/s11127-021-00897-8

[B81] Penfold E. D. Fourie P. (2015). Regional health governance: a suggested agenda for southern African health diplomacy. Glob. Soc. Policy 15 (3), 278–295. 10.1177/1468018115599817 26635498 PMC4639828

[B82] Rebai A. Abayomi A. Andanda P. Kerr R. Herbst K. Mabuka J. (2025). Responsible governance of genomics data and biospecimens in the context of broad consent: experiences of a pioneering access committee in Africa. BMJ Glob. Health 10 (2), e016026. 10.1136/bmjgh-2024-016026 PMC11808898 39922566

[B83] Robertson J. A. (2003). Procreative liberty in the era of genomics. Am. J. Law Med. 29 (4), 439–487. PubMed PMID: 15119245. 15119245

[B84] Roche (2025). African Genomics Program. Available online at: https://www.roche.com/stories/african-genomics-program (Accessed December 14, 2025).

[B85] Savulescu J. Kahane G. (2009). The moral obligation to create children with the best chance of the best life. Bioethics 23 (5), 274–290. 10.1111/j.1467-8519.2008.00687.x 19076124

[B86] Shozi B. Thaldar D. (2023). Promoting equality in the governance of heritable human genome editing through ubuntu: reflecting on a South African public engagement study. Am. J. Bioeth. 23 (7), 43–49. 10.1080/15265161.2023.2207524 37204147

[B87] Silver L. M. (1998). Special section: cloning: technology, policy, and ethics. Cloning, ethics, and religion. Available online at: https://www.leemsilver.net/SilverArticles/98CloningEthicsRel.pdf (Accessed November 6, 2025).

[B88] Ssebunnya G. M. (2023). Towards an appropriate African framework for public engagement with human genome editing: a call to synergistic action. Wellcome Open Res. 7, 302. 10.12688/wellcomeopenres.18579.2 37485292 PMC10359742

[B89] Staunton C. de Vries J. (2020). The governance of genomic biobank research in Africa: reframing the regulatory tilt. J. Law Biosci. 7 (1), lsz018. 10.1093/jlb/lsz018 34221433 PMC8249083

[B90] Stephan M. Marshall G. Mcginnis M. (2019). Governing Complexity. Cambridge University Press.

[B91] Stilgoe J. Lock S. J. Wilsdon J. (2014). Why should we promote public engagement with science? Public Underst. Sci. 23 (1), 4–15. 10.1177/0963662513518154 24434705 PMC5753839

[B92] Thaldar D. (2024). Editorial: data governance in African health research: ELSI challenges and solutions. Front. Pharmacol. 15, 1525434. PubMed PMID: 39723257. 10.3389/fphar.2024.1525434 39723257 PMC11668971

[B93] Thaldar D. Townsend B. Botes M. Shozi B. Pillay S. (2021). A virtual deliberative public engagement study on heritable genome editing among South Africans: study protocol. PLoS One 16 (8), e0256097. 10.1371/journal.pone.0256097 34411176 PMC8376038

[B94] Thaldar D. Shozi B. Steytler M. Hendry G. Botes M. Mnyandu N. (2022). A deliberative public engagement study on heritable human genome editing among South Africans: study results. PLoS One 17 (11), e0275372. 10.1371/journal.pone.0275372 36441783 PMC9704621

[B95] Thaldar D. Soni S. Prinsen L. Mnyandu N. Botes M. Kinderlerer J. (2025). Heritable human genome editing and the politics of law: the South African case study. J. Law Biosci. 12 (2), lsaf029. 10.1093/jlb/lsaf029 41445806 PMC12722161

[B96] Thaldar D. Amin N. Pillay S. Bruce-Brand J. Dlamini S. Govender R. (2026). Perspectives on south Africa’s open science policy: artificial intelligence and data ownership as unresolved questions. Sci. Public Policy, scag057. 10.1093/scipol/scag057

[B97] Tindana P. Yakubu A. Staunton C. Matimba A. Littler K. Madden E. (2019). Engaging research ethics committees to develop an ethics and governance framework for best practices in genomic research and biobanking in Africa: the H3Africa model. BMC Med. Ethics 20 (1), 69. 10.1186/s12910-019-0398-2 31623617 PMC6798385

[B98] Vaught J. (2017). “Biobanking in Africa: opportunities and challenges,” in Biobanking of Human Biospecimens (Cham: Springer International Publishing), 207–216. 10.1007/978-3-319-55120-3_12

[B99] Walker A. (2026). When genomics companies act as biobanks: governance challenges in an era of genomic assetization. Front. Genet. 17, 17. 10.3389/fgene.2026.1778236 PMC13016591 41890229

[B100] West African Genetic Medicine Centre (WAGMC) (2025). GhGenome. 1,000 Ghanaian genomes project. Available online at: https://wagmc.ug.edu.gh/ghgenome/1000-ghanaian-genomes-project/ (Accessed December 13, 2025).

[B101] WHO (2019). WHO’s global registry on human genome editing. Available online at: https://www.who.int/news/item/29-08-2019-who-launches-global-registry-on-human-genome-editing (Accessed June 22, 2026).

[B102] WHO (2024). Senegal and Rwanda achieve WHO maturity level 3 in medicines regulation. Available online at: https://www.who.int/news/item/05-12-2024-senegal-and-rwanda-achieve-who-maturity-level-3-in-medicines-regulation (Accessed June 22, 2026).

[B103] Wonkam A. (2021). Sequence three million genomes across Africa. Nature 590 (7845), 209–211. 10.1038/d41586-021-00313-7 33568829 PMC9979155

[B104] World Health Organization (2026). International clinical trials registry platform. Available online at: https://trialsearch.who.int (Accessed November 8, 2025).

[B105] World Health Organization (2021). WHO expert advisory committee on developing global standards for governance and oversight of human genome editing. Hum. Genome Ed. Position Pap.

[B106] World Health Organization (2025). Human genome editing registry. Available online at: https://www.who.int/groups/expert-advisory-committee-on-developing-global-standards-for-governance-and-oversight-of-human-genome-editing/registry (Accessed November 7, 2025).

[B107] Zou Y. Li Y. Tao Y. (2025). Regulatory framework of human germline and heritable genome editing in China: a comparison with the United States and the United Kingdom. J. Law Biosci. 12 (1), lsaf007. 10.1093/jlb/lsaf007 40364832 PMC12069028

